# Leaving the hospital on time: hospital bed utilization and reasons for discharge delay in the Netherlands

**DOI:** 10.1093/intqhc/mzad022

**Published:** 2023-04-07

**Authors:** Eva van den Ende, Bo Schouten, Lara Pladet, Hanneke Merten, Louise van Galen, Milka Marinova, Michiel Schinkel, Anneroos W Boerman, Rishi Nannan Panday, Cees Rustemeijer, Muhammad Dulaimy, Derek Bell, Prabath Wb Nanayakkara

**Affiliations:** Section General Internal Medicine Unit Acute Medicine, Department of Internal Medicine, Amsterdam Public Health Research Institute, Amsterdam University Medical Center, Location VU University Medical Center, De Boelelaan 1117, Amsterdam 1081 HV, The Netherlands; Department of Public and Occupational Health, Amsterdam Public Health Research Institute, Amsterdam University Medical Center, Location VU University Medical Center, De Boelelaan 111, Amsterdam 1081 HV, The Netherlands; Section General Internal Medicine Unit Acute Medicine, Department of Internal Medicine, Amsterdam Public Health Research Institute, Amsterdam University Medical Center, Location VU University Medical Center, De Boelelaan 1117, Amsterdam 1081 HV, The Netherlands; Department of Public and Occupational Health, Amsterdam Public Health Research Institute, Amsterdam University Medical Center, Location VU University Medical Center, De Boelelaan 111, Amsterdam 1081 HV, The Netherlands; Section General Internal Medicine Unit Acute Medicine, Department of Internal Medicine, Amsterdam Public Health Research Institute, Amsterdam University Medical Center, Location VU University Medical Center, De Boelelaan 1117, Amsterdam 1081 HV, The Netherlands; Imperial College London, Lift Bank D, Chelsea and Westminster Hospital, NHS Foundation Trust, 369 Fulham Road, London SW10 9NH, United Kingdom; Section General Internal Medicine Unit Acute Medicine, Department of Internal Medicine, Amsterdam Public Health Research Institute, Amsterdam University Medical Center, Location VU University Medical Center, De Boelelaan 1117, Amsterdam 1081 HV, The Netherlands; Center for Experimental and Molecular Medicine (CEMM), Amsterdam UMC, Location Academic Medical Center, Meibergdreef 9, Amsterdam 1105 AZ, The Netherlands; Section General Internal Medicine Unit Acute Medicine, Department of Internal Medicine, Amsterdam Public Health Research Institute, Amsterdam University Medical Center, Location VU University Medical Center, De Boelelaan 1117, Amsterdam 1081 HV, The Netherlands; Department of Clinical Chemistry, Amsterdam UMC, Location VU University Medical Center, De Boelelaan 1118, Amsterdam 1081 HZ, The Netherlands; Section General Internal Medicine Unit Acute Medicine, Department of Internal Medicine, Amsterdam Public Health Research Institute, Amsterdam University Medical Center, Location VU University Medical Center, De Boelelaan 1117, Amsterdam 1081 HV, The Netherlands; Department of Internal Medicine, Amstelland Hospital, Laan van de Helende Meesters 8, Amstelveen 1186 AM, The Netherlands; Department of Internal Medicine, Zaans Medical Center, Koningin Julianaplein 58, Zaandam 1502 DV, The Netherlands; Imperial College London, Lift Bank D, Chelsea and Westminster Hospital, NHS Foundation Trust, 369 Fulham Road, London SW10 9NH, United Kingdom; Section General Internal Medicine Unit Acute Medicine, Department of Internal Medicine, Amsterdam Public Health Research Institute, Amsterdam University Medical Center, Location VU University Medical Center, De Boelelaan 1117, Amsterdam 1081 HV, The Netherlands

**Keywords:** delays in care, acute hospital, The Netherlands, delayed discharges, Day of Care Survey, inpatient flow, inappropriate stay

## Abstract

Inappropriate bed occupancy due to delayed hospital discharge affects both physical and psychological well-being in patients and can disrupt patient flow. The Dutch healthcare system is facing ongoing pressure, especially during the current coronavirus disease pandemic, intensifying the need for optimal use of hospital beds. The aim of this study was to quantify inappropriate patient stays and describe the underlying reasons for the delays in discharge. The Day of Care Survey (DoCS) is a validated tool used to gain information about appropriate and inappropriate bed occupancy in hospitals. Between February 2019 and January 2021, the DoCS was performed five times in three different hospitals within the region of Amsterdam, the Netherlands. All inpatients were screened, using standardized criteria, for their need for in-hospital care at the time of survey and reasons for discharge delay. A total of 782 inpatients were surveyed. Of these patients, 94 (12%) were planned for definite discharge that day. Of all other patients, 145 (21%, ranging from 14% to 35%) were without the need for acute in-hospital care. In 74% (107/145) of patients, the reason for discharge delay was due to issues outside the hospital; most frequently due to a shortage of available places in care homes (26%, 37/145). The most frequent reason for discharge delay inside the hospital was patients awaiting a decision or review by the treating physician (14%, 20/145). Patients who did not meet the criteria for hospital stay were, in general, older [median 75, interquartile range (IQR) 65–84 years, and 67, IQR 55–75 years, respectively, *P* < .001] and had spent more days in hospital (7, IQR 5–14 days, and 3, IQR 1–8 days respectively, *P* < .001). Approximately one in five admitted patients occupying hospital beds did not meet the criteria for acute in-hospital stay or care at the time of the survey. Most delays were related to issues outside the immediate control of the hospital. Improvement programmes working with stakeholders focusing on the transfer from hospital to outside areas of care need to be further developed and may offer potential for the greatest gain. The DoCS can be a tool to periodically monitor changes and improvements in patient flow.

## Introduction

Pressure on hospital beds is a common problem in healthcare systems [1]. Over the past 2 years, this problem has intensified as COVID-19 patients added additional pressure on the hospital bed availability, contributing to a shortage in healthcare personnel as the pandemic evolved [2].

One approach to dealing with the shortage of beds is by reducing avoidable delays in the discharge process, thereby minimizing the number of patients occupying a bed without a clinical indication [3]. Discharge delays lead to congestion across the healthcare chain, resulting in overcrowding and sometimes forcing closure of emergency departments or other acute facilities [4]. For inpatients, prolonged hospital stays are associated with increased risk of adverse events such as nosocomial infections, hospital-acquired delirium, increased dependency, loss of muscle mass, and even death [5–9]. Both patient and caregiver experiences are negatively influenced by discharge delays [10], which contributes directly to increasing healthcare costs [11, 12].

Information about the extent of inappropriate hospital bed utilization and the associated underlying reasons in the Netherlands is scarce and outdated. Two Dutch studies (published in 2002 and 2003) reported percentages of inappropriate hospital stay of 20% and 21%, respectively [1, 13]. Due to recent policy changes in the healthcare system and concomitant changes in case mix, these percentages are no longer up to date [14, 15]. Identifying current factors contributing to discharge delays will help develop specific improvement programmes to optimize patient flow and permit ongoing monitoring to support interventions.

The Day of Care Survey (DoCS) provides a cross-sectional overview of a hospital’s inappropriate bed occupancy and identifies issues inside and outside the hospital contributing to discharge delay. The DoCS is validated and has been used to evaluate over 10 000 patients across Australia and the UK [16]. The survey was developed by Reid et al. in 2011 and is based on the widely used appropriateness evaluation protocol, which focuses on inappropriate days spent in the hospital [17]. The aim of this study was to quantify discharge delays and identify underlying reasons by means of this internationally used and validated DoCS [16].

## Methods

This study was conducted between February 2019 and January 2021 and consisted of five cross-sectional surveys. One academic hospital was surveyed three times (February 2019, November 2019, and January 2021), and two non-academic hospitals each once (October 2019 and January 2020). All hospitals were located in the region of Amsterdam, the Netherlands, and had a capacity between 141 and 288 beds on the days of survey. Surveys were performed on weekdays between 9 a.m. and 11 a.m., excluding Mondays and Fridays to avoid potential confounding factors related to bed occupancy at weekends. All adult (≥18 years) inpatients, excluding those admitted to the intensive care and high dependency units, mental health, and maternity wards, were screened using the DoCS. Patients who spent over 4 hours in the emergency department awaiting admission were considered inpatients, as they would likely require hospital admission. Due to differences in ward structures between the included hospitals, patients were subdivided into four categories: inpatient medicine, inpatient surgery, emergency services, and acute services. Patients who were already planned for definite discharge at the time of survey were excluded from further analysis.

The procedures were organized according to the original National Health Service (NHS) protocol for the DoCS [16]. First, an expert team of medical specialists in internal medicine approved the applicability of the protocol for the Dutch health system and also checked and corrected the Dutch translation of the survey. Only minor changes in nomenclature regarding reasons for inpatient delay were applied to fit into the Dutch healthcare system (e.g. ‘disagreement between family/patient/NHS/local authority’ was changed to ‘disagreement between family/patient/hospital/insurance/local authority’). No structural adjustments were made to the protocol. Second, the principal investigator of the DoCS in the UK (Dr Milka Marinova) was invited to the Netherlands to train two local researchers (Dr Eva van den Ende [medical doctor] and Bo Schouten [psychologist]) in conducting the survey, analysing the data, and supervising the pilot survey. The local researchers acted as overarching co-ordinators of the project. Additionally, a local co-ordinator in each hospital was assigned. The hospital board and department heads were informed about the study and the protocol used but did not receive in-depth information regarding the procedures or the methods to avoid information bias. On the morning of the survey, local surveyors were paired into teams and instructed on how to use the DoCS. Each team consisted of a medical doctor and a second healthcare professional (nurse, manager, medical student, or another doctor). Teams did not survey their own departments. The overarching research team did not have access to the patient records and did not see the patient, and no identifiable data were shared and/or collected.

### Data collection

All patients in the participating wards discussed with their designated senior nurse with the aim of determining whether they met the criteria for inpatient care. The senior nurse consulted patient records when necessary. Each patient was assessed against a range of 28 clinical criteria warranting inpatient care according to the DoCS (Supplementary eTable 1). These criteria could be signs of severe illness (such as a systolic blood pressure < 90 mmHg) or the need for services that required hospital facilities (such as frequent vital sign check-ups or oxygen therapy). The team could overrule the criteria in either direction, based on clinical judgement, if deemed necessary (e.g. an athlete with a low resting pulse rate of <50 bpm without meeting any other criteria for hospitalization). If a patient checked one or more of the clinical criteria, the hospital stay was classified as ‘appropriate’. No further information concerning the number or nature of the criteria was collected. If a patient did not meet a single criterion, the stay was classified as ‘inappropriate’, and the reason for the discharge delay and an alternative place of care were specified. Reasons for discharge delay were subdivided into inside hospital issues (such as awaiting consultation by the treating physician) and outside hospital issues (such as awaiting availability for home care). If there was more than one factor delaying discharge, the major issue preventing the patient from leaving the hospital was recorded. Lastly, all ‘boarders’ (patients occupying a bed on a ‘wrong’ ward due to bed shortages on the ward of their specialty, also known as outliers) were marked.

All data were initially recorded on a standardized data collection sheet and then directly entered into a programmed Excel sheet.

### Data analysis

Microsoft Office Excel (2016) was used for data recording, and SPSS for Windows (version 26) was used for data analysis. Descriptive statistics were performed. Categorical variables are summarized by percentages, and continuous data are summarized by the median and interquartile range (IQR) after checking for normality.

## Results

In the five surveys, a total of 1055 available beds with 782 patients were screened. Findings showed 169 (16%) closed beds due to, e.g., staff shortages and an overall hospital bed occupancy of 88% (ranging from 77% to 96%). A total of 94 (12%) patients were planned for definite discharge on the day of the survey and were excluded from further analysis. Of the remaining patients, 145 (21%, range 14%–35%) did not meet the DoCS criteria for acute in-hospital care and 47 (6%) were boarders (Table 1).

**Table 1.** Summary of results of five Day of Care Surveys across three hospitals.

Survey number	1	2	3	4	5	Total
Survey date	February 2019	October 2019	20 November 2019	January 2020	January 2021	
Hospital^a^	A1	NA2	A1	NA3	A1	
Total number of beds on the day of the survey^b^	244	141	222	160	288	1055
Total number of patients surveyed	208	100	167	133	174	782
Patients being discharged on the day of survey	25	10	24	17	18	94
Bed occupancy^c^	96%	91%	89%	90%	77%	88%
Closed beds^d^	12% (28)	22% (31)	16% (35)	8% (13)	22% (62)	16% (169)
Empty beds	6% (15)	7% (10)	13% (28)	11% (17)	18% (52)	12% (122)
Boarders^e^	3% (7)	4% (4)	6% (10)	8% (13)	8% (13)	6% (47)
DoCS criteria^f^						
Met	81% (149)	80% (71)	79% (114)	65% (75)	87% (135)	79% (544)
Not met	19% (34)	21% (19)	21% (30)	35% (41)	14% (21)	21% (145)
Inside hospital issues	35% (12)	16% (3)	23% (7)	20% (8)	19% (4)	23% (34)
Outside hospital issues	62% (21)	84% (16)	77% (23)	73% (30)	81% (17)	74% (107)
Unknown	3% (1)	0% (0)	0% (0)	7% (3)	0% (0)	3% (4)
Patient characteristics						
Age in years	68 (55–76)	76 (65–83)	64 (51–72)	73 (66–82)	67 (55–76)	69 (57–78)
Age DoCS criteria ‘met’	65 (53–73)	73 (64–83)	64 (51–71)	71 (63–79)	66 (55–75)	67 (55–75)
Age DoCS criteria ‘not met’	76 (70–87)	82 (74–89)	64 (56–72)	79 (71–87)	66 (44–79)	75 (65–84)
LOS in days	6 (2–12)	2 (1–7)	4 (1–7)	3 (1–9)	4 (1–7)	4 (1–9)
LOS DoCS criteria ‘met’	6 (2–13)	2 (1–3)	4 (1–7)	2 (1–5)	3 (1–7)	3 (1–8)
LOS DoCS criteria ‘not met’	6 (3–11)	9 (7–14)	7 (4–13)	6 (4–15)	4 (2–22)	7 (5–14)

All data are presented as n, % (n), or median (IQR). ^a^Academic hospital (A)/non-academic (NA) + serial number. ^b^Including closed beds. ^c^Excluding closed beds. ^d^Closed beds due to shortage of staff or budgetary cuts. ^e^Patients occupying a bed on a ‘wrong’ ward due to bed shortages on the ward of his/her specialty. Percentages of the total number of patients surveyed. ^f^Percentages of all surveyed patients excluding those who are discharged on the day.

### Age

**Figure 1:**
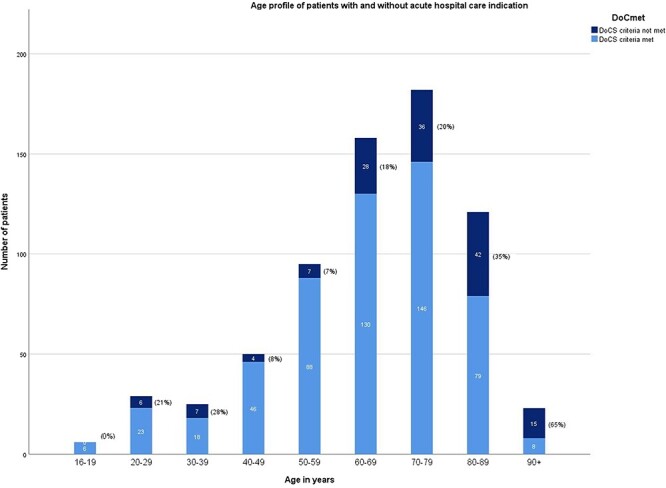
Age profile of patients with and without an acute in-hospital care indication.

The median age of all admitted patients was 69 years (IQR 57–78 years). The median age of patients in the two non-academic hospitals (76 and 73 years, respectively) was higher than that in the academic hospital (68, 64, and 67 years, respectively) in all three surveys. Patients who did not meet the criteria for acute in-hospital care had higher median age than those who did (75 years, IQR 65–84 years; 67 years, IQR 55–75 years, respectively, *P* < .001) (Table 1). Of all surveyed patients, 47% (326/688) were 70 years or older. The majority of patients (64%, 93/145) not meeting the criteria were over 70 years old (Figure 1).

### Length of stay

**Figure 2:**
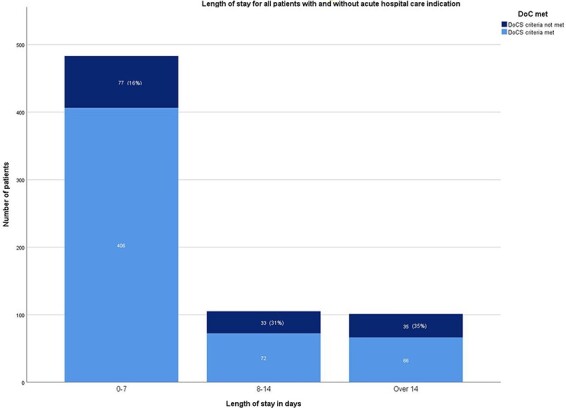
LOS for all patients with and without an acute clinical indication for in-hospital care.

The median length of stay (LOS) at the time of the survey was 4 days (IQR 1–9 days). Patients who did not meet the criteria had a median LOS twice as long as that of patients who did meet the criteria for acute in-hospital care (7, IQR 5–14 days; 3, IQR 1–8 days, respectively) (Table 1). Most patients (70%, 483/689) had spent 0 to 7 days in the hospital at the time of survey. These patients were half as likely to occupy a bed without an acute indication compared to patients in the groups with a longer LOS (LOS 0–7 days, 16%; 8 to 14 days, 31%; and over 14 days, 35%; Figure 2). The percentage of patients with a LOS of over 8 days varied (range 20%–42%) (Supplementary eFigure 1).

### Ward specialty

**Figure 3:**
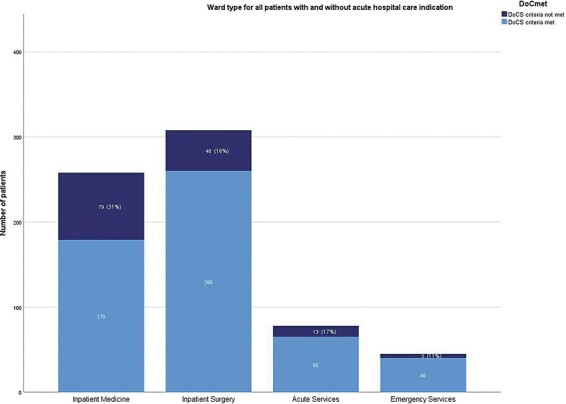
Ward type for all patients with and without an acute in-hospital care indication.

The wards were subdivided into four categories: inpatient medicine, inpatient surgery, acute services, and emergency services. Inpatient medicine patients had the highest proportion of patients without an acute clinical indication for in-hospital care (31%). This was almost twice as high as in the remaining groups (16%, 17%, and 11%, respectively) (Figure 3).

### Reasons for delay

**Figure 4:**
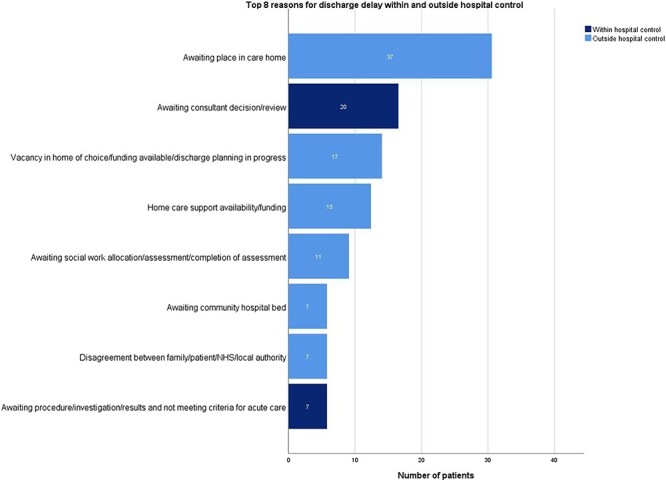
The top eight reasons for discharge delays, both inside and outside of hospital control.

A list of all 20 reasons for delay can be found in Supplementary eFigure 2. Reasons for discharge delay were divided into two categories: delays due to inside hospital issues (23%) and outside hospital issues (74%). In 3%, the reason for delay was unclassified. In all surveys, most delays were due to outside hospital issues (range 62%–84%) (Table 1). Figure 4 shows the top eight reasons of discharge delay, covering 83% (121/145) of all recorded reasons. The most frequent reasons for delay were a shortage of available places in care homes (26%, 37/145) (outside hospital issue) and ‘awaiting decision or review by the treating physician’ (14%, 20/145) (inside hospital issue). For patients under 60 years old, ‘awaiting consultant decision or review’ was the most frequent reason for delay, whereas for patients over 60 years of age, it was ‘waiting for a place in a care home’ (Supplementary eFigure 3). Of patients awaiting a place in a care home, 70% (26/37) were on a medical ward (Supplementary eFigure 4). In non-academic hospitals, awaiting a place in a care home was almost four times more often the reason for delay than the second most common reason ‘awaiting consultant decision’ (38% and 10%, respectively). In the academic hospital, the reasons were equally common (awaiting a place in care home 16% and awaiting consultant decision 16%) (Supplementary eFigure 5). The ratio of outside/inside hospital issues did not significantly change during the COVID-19 pandemic (academic pre-COVID-19: Survey 1, 62% outside; Survey 3, 84% outside; academic during COVID-19: Survey 5, 81% outside; *P* = .333). Supplementary eFigure 6 shows the reasons for discharge delay for each survey.

### Alternative place of care

The appropriate place of care for delayed patients was found to be in a non-acute area of care (e.g. a care home) in 60% and at home (with or without home care) in 35% of patients (Supplementary eFigure 6).

## Discussion

### Statement of principal findings

In this study, we found that almost one in five inpatients were inappropriately utilizing beds according to the DoCS criteria. The eight most common reasons for delay accounted for 83% of all recorded reasons, with awaiting a place in a care home being the most frequent (one in four delays). The second most frequent reason was ‘awaiting the consultant’s decision or review’, which was more common in academic hospitals (16%) than in non-academic hospitals (10%), possibly due to organizational differences (e.g. the physical presence of consultant physicians on the wards) [18]. This is important to address, as internal hospital issues may provide quicker gains through improving decision-making pathways alone.

### Strengths and limitations

The DoCS has been internationally used and has proven to be a comprehensive and quick methodology to assess discharge delays, which can be performed in a short period of time with little disruption for the healthcare staff [16]. Data can easily be compared between centres and between countries with similar healthcare systems. The researchers conducting this study were well trained to successfully use the survey. However, this study has some limitations. First, the information provided is a ‘snapshot’, showing the situation at a particular moment in time. Our data were gathered over a period of almost 2 years to account for changes in patient flow. Results did not show great variation between survey times in this study (even with the onset of the COVID-19 pandemic during which Survey 5 was carried out) and with surveys performed in other countries. The risk of bias was therefore assumed to be low. Second, for each patient only the first reason for delay was documented. It is possible that for some patients, after the decision by the treating physician had been made, the next blockage would arise, such as finding a place in a care home. Therefore, the magnitude of the transition problems from hospital to long-term community care might still be underestimated in this study.

### Interpretation within the context of the wider literature

The published literature shows that the DoCS have previously been applied in 23 London hospitals (Marinova et al.) and nine hospitals across Scotland and England (Reid et al.) [16, 19]. A comparable percentage of delayed discharges was found (current study 21%, Reid et al. 23%, and Marinova et al. 24%). However, this study revealed lower occupancy levels in the Netherlands (average 88%) than in the London hospitals in the study of Marinova et al. (average 97%). Possibly as a result, we found that the reasons for discharge delay were less frequently caused by issues inside the hospital (current study 23% and Marinova et al. 34%). In contrast, difficulties in transferring patients to outside areas of care seem to be a bigger problem in the Netherlands (current study 74% and Marinova et al. 61%) [16, 19].

The percentage of inappropriate stays in this study is similar to the percentage of inappropriate days of care in two previous Dutch studies (20% and 21%) [1, 13]. This suggests no significant overall change over the past 20 years. However, delays seem to be less frequently caused by inside hospital issues presently, possibly due to organizational changes such as the implementation of electronic health records [10]. The reason for delay has shifted more towards difficulties transferring inpatients to outpatient areas of care (delays caused by issues outside the hospital; previous Dutch study 55% and current study 74%) [1]. This may be an effect of the growing need for (chronic) care due to an increase in chronic illness, ageing of the population, and less capacity in care homes and home care due to cross-wide healthcare chain staff shortages [20–22]. Furthermore, the Dutch healthcare system was reformed in 2015, stimulating older people to live self-dependently at home for a longer period of time by closing nursing homes and tightening up the criteria for admittance [21]. Consequently, more patients are admitted from home, cannot return to their previous home situation after discharge, and need to wait for a place in a care home (45).

### Implications for policy, practice, and research

The first DoCS study of Reid et al. highlighted that older patients and those with a longer LOS were more likely to experience discharge delays [16]. Although we found that discharge delay was indeed more common in older patients (60–100 years), our results showed similar percentages of delayed patients in the age bands up to 60 years. As in both groups, other types of exit blocks dominate (‘awaiting the consultant’s decision or review’ and ‘awaiting a place in a care home’, respectively), targeting only older patients to tackle discharge delays would miss opportunities relevant to all patients and the system. With regard to LOS, we recognized the trend described in earlier research: delays increase with increasing LOS [16, 19]. However, half (53%) of all delayed patients in this study were in the 0–7 LOS band. A single focus on patients with a longer LOS would be insufficient; instead, a needs-based approach would be more appropriate.

As in previous DoCS studies, patients on medical wards were most likely to suffer from discharge delay (31%) [16, 19]. In comparison to surgical patients, medical patients are often older and suffer from more complex medical, psychological, and social problems [23, 24]. In addition, most surgical patients have integrated care pathways (e.g. after hip replacement), making it easier to transfer the patient to a predetermined place [25]. Nevertheless, one in six surgical patients were labelled as delayed.

Delays due to outside hospital issues could potentially be reduced by early discharge planning and improving communication pathways between hospitals and community care. Coffey et al. suggested a shared and centralized electronic patient record system and insight into care capacity [26]. Advanced care planning and increasing the number and capacity of initiatives such as ‘first line stays’ (temporary beds for patients without an indication for admission to the hospital but who cannot immediately return to their homes), ‘hospital at home’ (hospital care in the home situation in order to prevent hospital admissions and related complications) as well as regular care homes [27, 28] are required. Delays caused by awaiting decision or review by the treating physician could potentially be improved by reviewing the process by which this is done to improve efficiency. After implementation of improvement programmes, we recommend DoCS as a useful tool to monitor change and analyse progress in patient flow.

Future research should focus on the factors complicating the transfer from hospital to outside areas of care in the Netherlands; for example, shortages in care home availability and difficulties in hospital-care home communication [29, 30].

## Conclusions

The prevalence of inappropriate bed occupation in three Dutch hospitals was 21% (range 13%–35%). Patients who experienced discharge delays were more often admitted to a medical ward, were older, and had a longer LOS. One in four delayed patients were awaiting a place in a care home, making this the most frequent reason for delay. The second most frequent reason for delay was awaiting a consultant’s decision or review. Three out of four delays were caused by issues outside the hospital. Therefore, interventions to reduce discharge delays should focus on improving the transfer from the hospital to successive links in the care chain.

## Supplementary Material

mzad022_Supp

## Data Availability

The dataset is available after the approval of the corresponding author.
