# Supplementary material for: Leaving the hospital on time: hospital bed utilization and reasons for discharge delay in the Netherlands
Source: Int J Qual Health Care. 2023 Apr 7;35(2):mzad022. doi: 10.1093/intqhc/mzad022 (PMC10411855; doi:10.1093/intqhc/mzad022)

# Supplementary content

**eTable 1.** Criteria for appropriate bed utilization

**eFigure 1.** Length of stay for in 7 day intervals for each survey moment

**eFigure 2.** All reasons for discharge delay

**eFigure 3.** Reasons for discharge delay for different age bands

**eFigure 4.** Reasons for discharge delay for different departments

**eFigure 5.** Reasons for discharge delay for academic and community hospitals

**eFigure 6.** Reasons for discharge delay for each survey

**eFigure 7.** Alternative places of care for patients with an inappropriate hospital stay

## eTable 1. Criteria for appropriate bed utilization

| **Service intensity that requires access to acute hospital inpatient facilities** | | |
| --- | --- | --- |
| 1 |  | Acute or ongoing deterioration in conscious level |
| 2 |  | Acute or ongoing new confusion |
| 3 |  | Acute neurological deficit, including stroke within 72 hours |
| 4 |  | Acute coronary syndrome confirmed or suspected |
| 5 |  | Acute dysrhythmia with haemodynamic disturbance |
| 6 |  | Pulse rate <50 or >100 bpm |
| 7 |  | BP systolic <90 mmHg |
| 8 |  | Phase IV hypertension |
| 9 |  | Active bleeding |
| 10 |  | Transfusion due to blood loss |
| 11 |  | Temperature <35°C or >38°C |
| 12 |  | Arterial pH <7.3 or pH >7.45 |
| 13 |  | Na <123 or >150 mmoSl/l |
| 14 |  | K <2.5 or >6.0 mmol/l |
| 15 |  | Acute kidney injury |
| 16 |  | Post-operative ileus |
| **Service intensity that requires access to acute hospital inpatient facilities** | | |
| 17 | Therapy | Requires IV, IM or subcutaneous medication (that cannot be delivered at home/in the community) |
| 18 | Therapy | Receiving treatment or new/experimental treatment requiring frequent dose adjustments or medical monitoring under direct medical supervision |
| 19 | Procedure | Surgical procedure today that is not suitable for day case |
| 20 | Procedure | Invasive procedure not suitable for day case (eg some interventional radiology, some guided biopsies etc) |
| 21 | Monitoring | Vital sign monitoring every hour or more frequently |
| 22 | Monitoring | Chemotherapy requiring constant supervision |
| 23 | Monitoring | Requires accurate input/output fluid balance measurement |
| 24 | Respiratory | Requires continuous oxygen, non-invasive ventilation or intensive nebuliser therapy that cannot be delivered at home |
| 25 | Fluid/nutrition | To establish complex nutritional support, including enteral feeding |
| 26 | Fluid/nutrition | Requires intravenous fluids (that cannot be delivered at home/in the community) |
| 27 | Recovery | Immediate postoperative recovery phase from therapy/procedure covered in 19 and 20 (above), including need for complex dressings/wound drainage (that cannot be delivered in the community/at home) |
| 28 | Investigation | Requires multiple investigations for urgent diagnosis |

If a patient checks one or more of the criteria to stay in hospital, the patient is classified as ‘appropriate’

## eFigure 1. Length of stay for in 7 day intervals for each survey moment


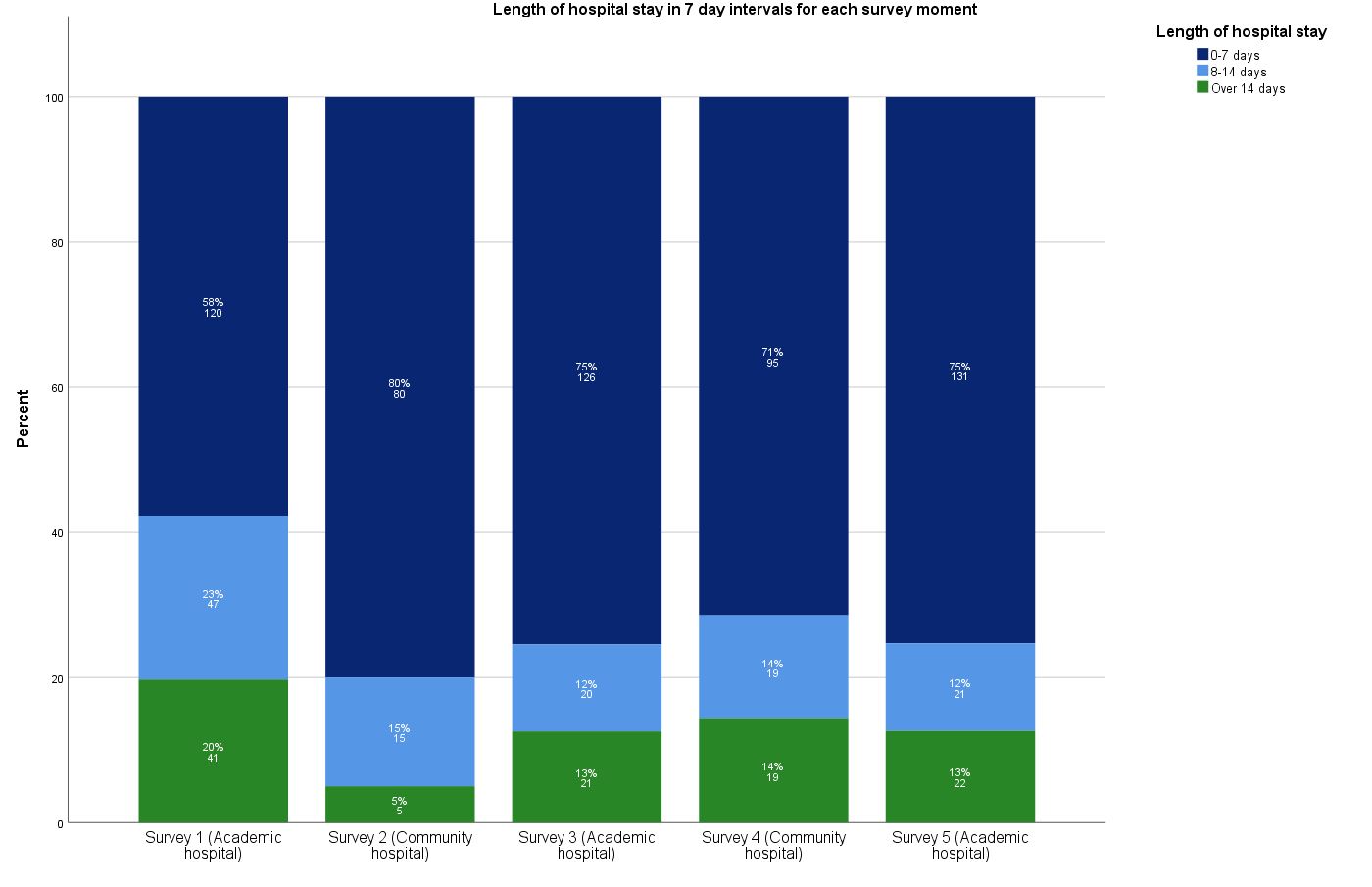


## eFigure 2. All reasons for discharge delay


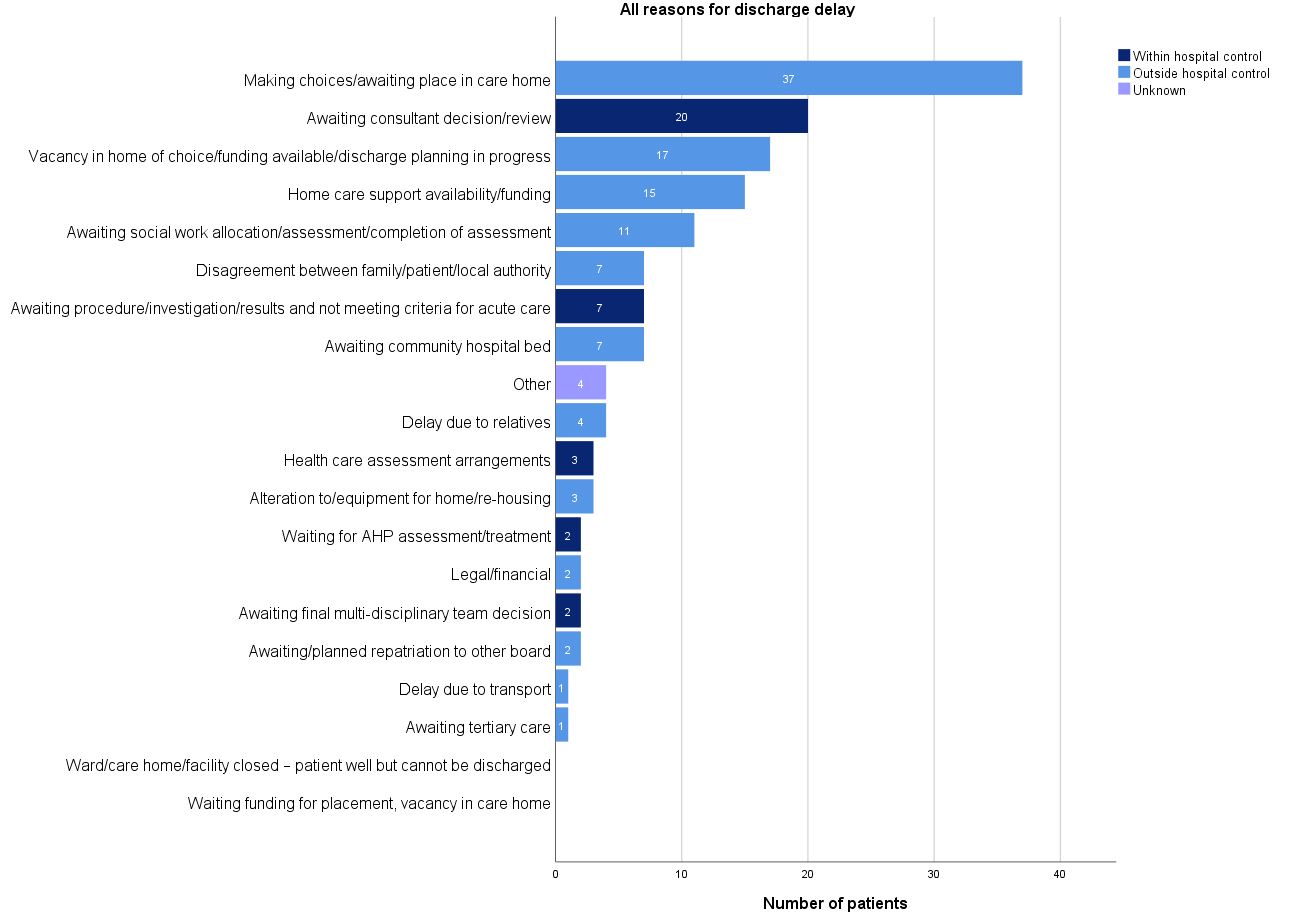


## eFigure 3. Reasons for discharge delay for different age bands


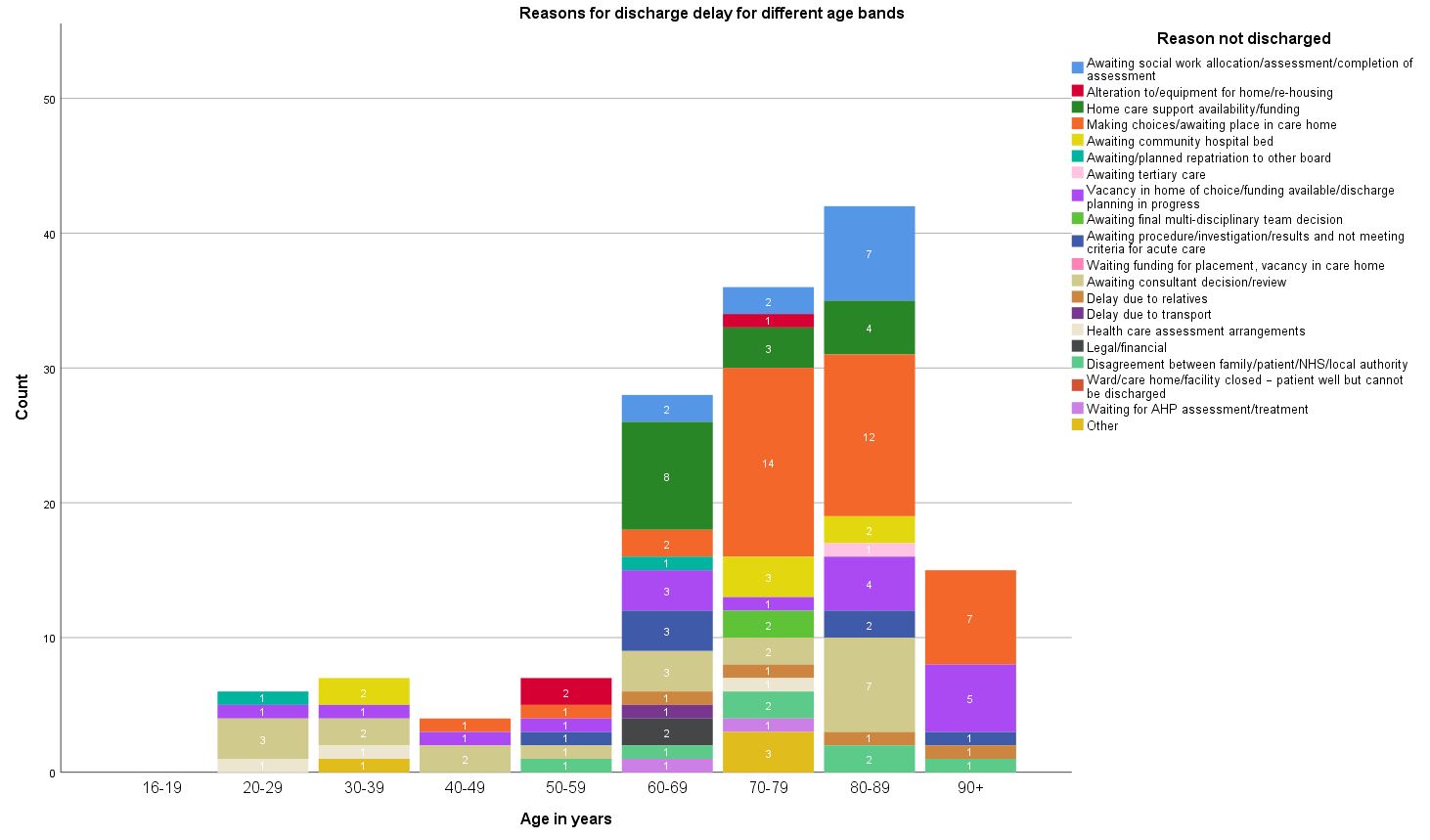


## eFigure 4. Reasons for discharge delay for different departments


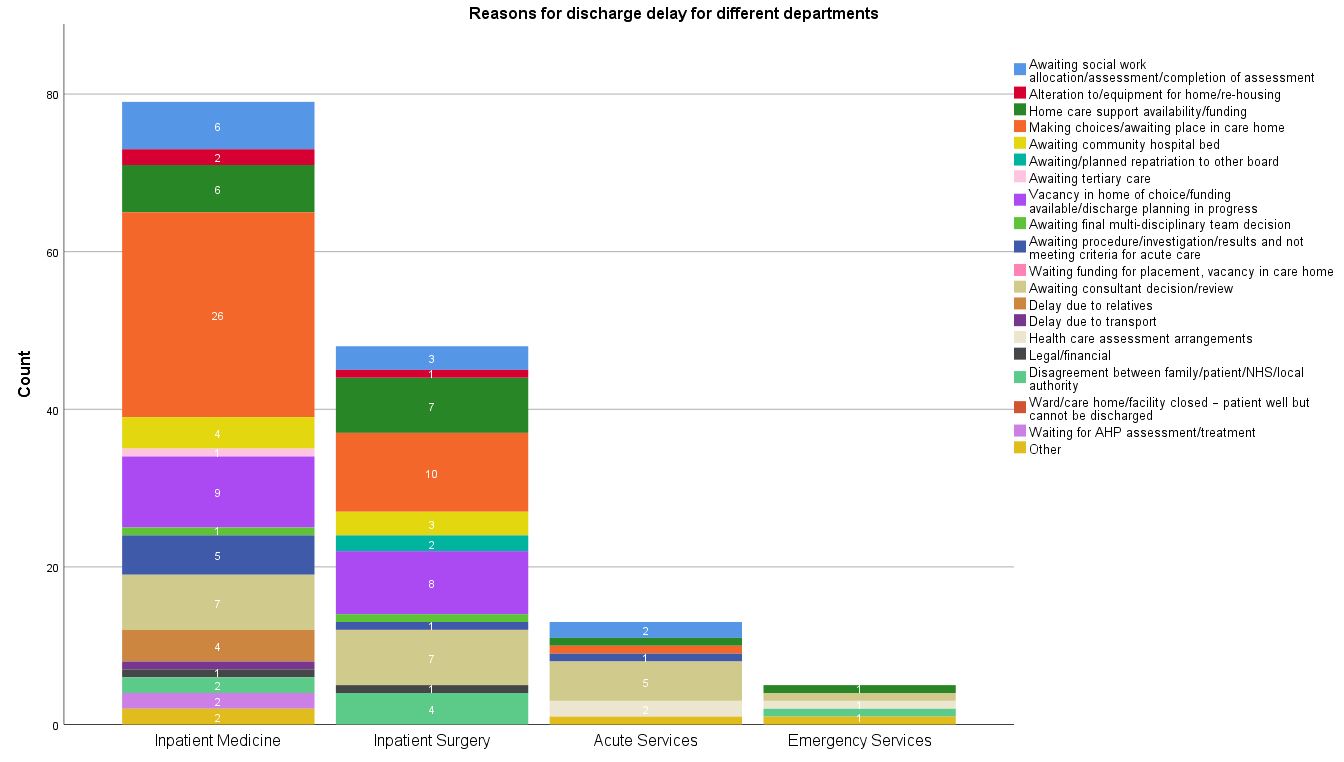


## eFigure 5. Reasons for discharge delay for academic and community hospitals


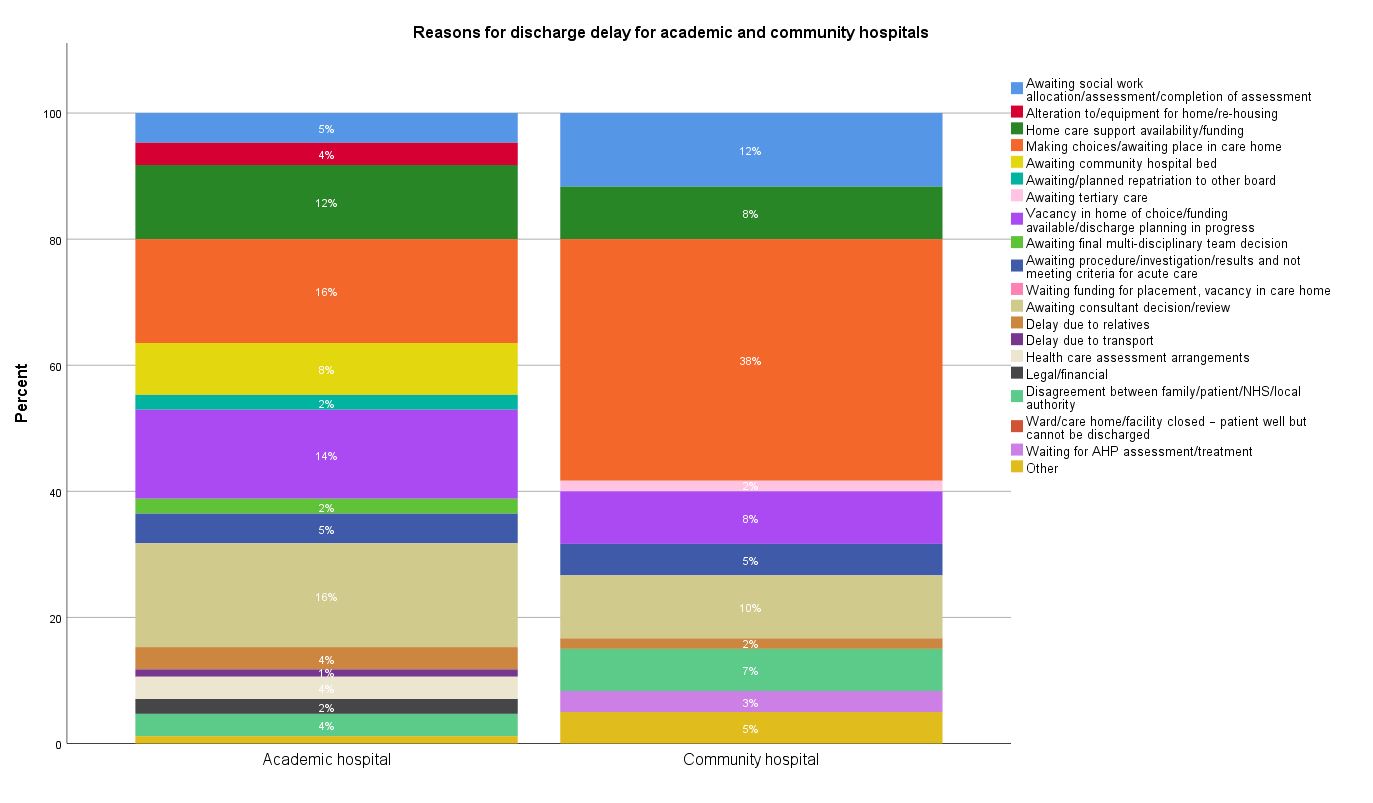


## eFigure 6. Reasons for discharge delay for each survey


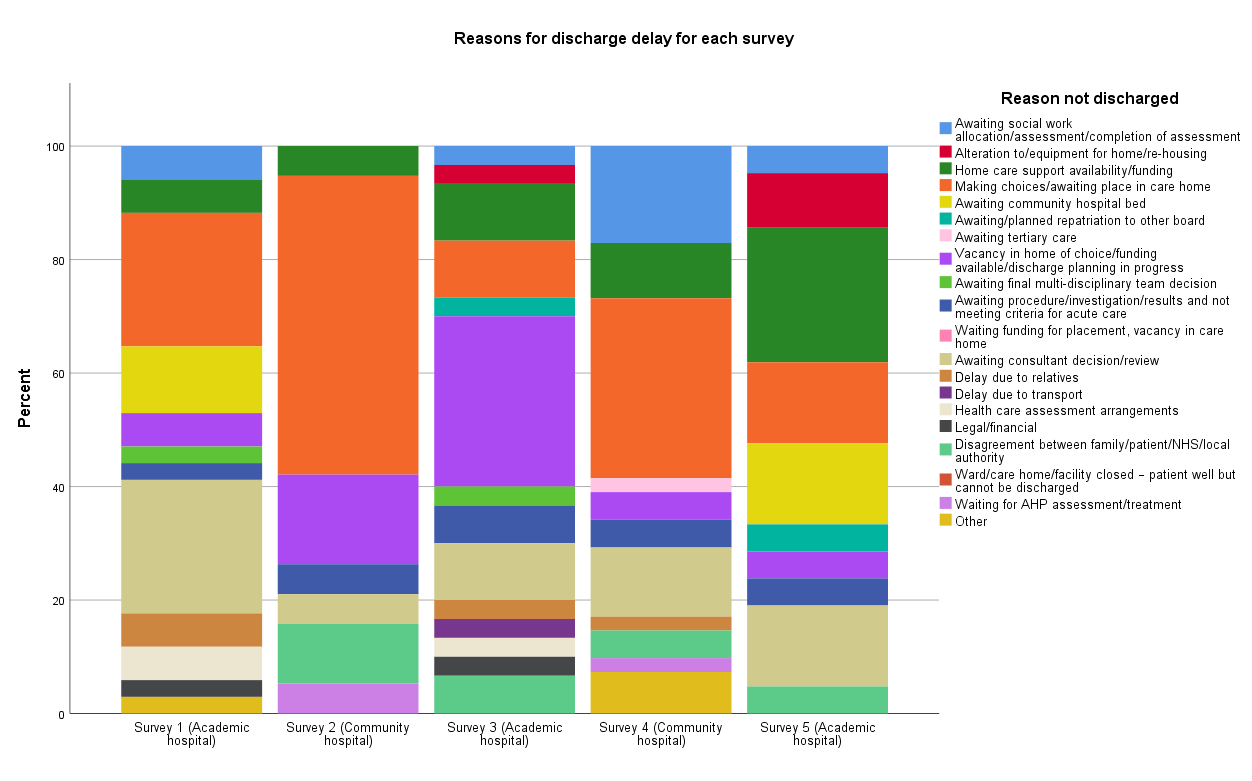


## eFigure 7. Alternative places of care for patients with an inappropriate hospital stay


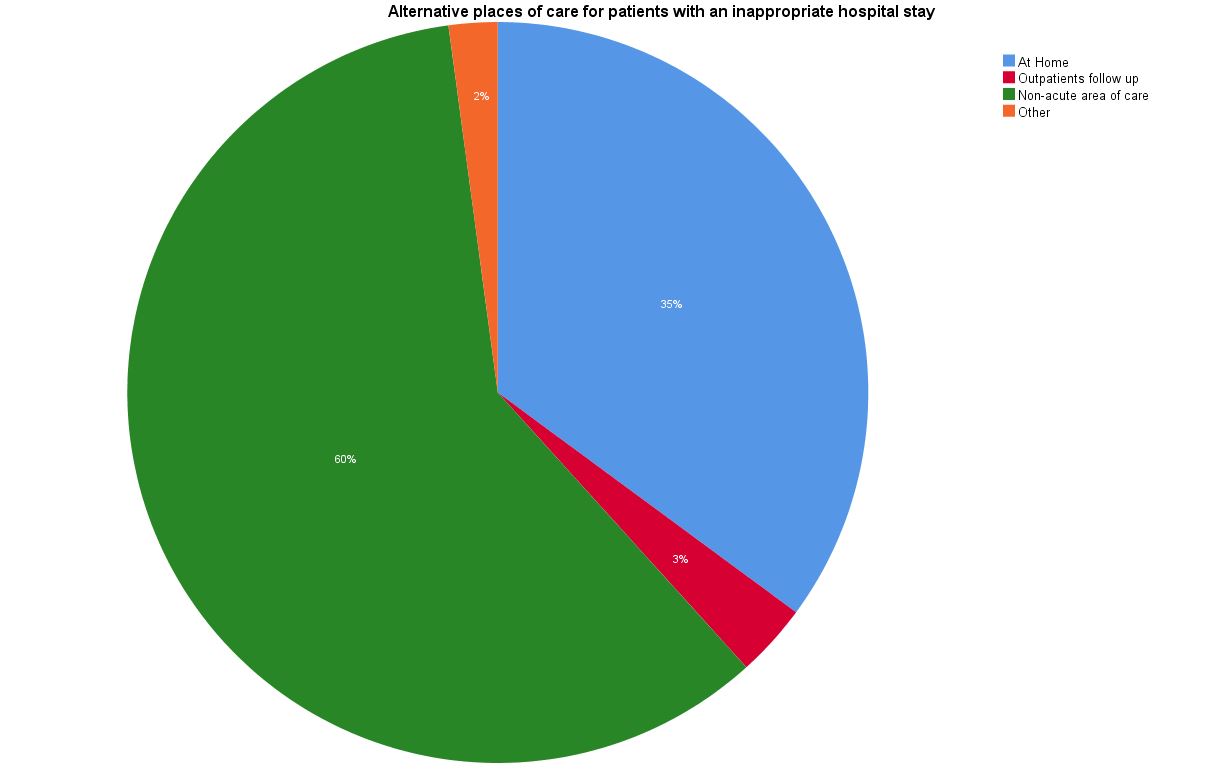

Supplement: mzad022_Supp [file mzad022_supp.zip › suppl_data/DoCS_Supplement.docx]
